# A global proficiency testing programme for Xpert® MTB/RIF using dried tube specimens, 2013–2015

**DOI:** 10.4102/ajlm.v9i1.1167

**Published:** 2020-11-27

**Authors:** Katherine Klein, Kyle DeGruy, Zilma Rey, Patricia Hall, Andrea Kim, Steve Gutreuter, Heather Alexander

**Affiliations:** 1 Centers for Disease Control and Prevention, National Center for HIV/AIDS, Viral Hepatitis, STD and TB Prevention, Division of TB Elimination, Atlanta, Georgia, United States; 2 Centers for Disease Control and Prevention, Division of Global HIV and TB, International Laboratory Branch, Atlanta, Georgia, United States; 3 Centers for Disease Control and Prevention, Division of Global HIV and TB, Epidemiology and Surveillance Branch, Atlanta, Georgia, United States; 4 Centers for Disease Control and Prevention, Division of Global HIV and TB, Health Informatics, Data Management and Statistics Branch, Atlanta, Georgia, United States

**Keywords:** external quality assessment, Xpert® MTB/RIF, proficiency testing, dried tube specimens, resource-limited settings

## Abstract

**Background:**

Proficiency testing (PT) is an important quality assurance measure toward ensuring accurate and reliable diagnostic test results from clinical and public health laboratories. Despite the rapid expansion of the Xpert® MTB/RIF assay for the detection of tuberculosis in resource-limited settings (RLS), low-cost PT materials for Xpert MTB/RIF external quality assessment (EQA) are not widely available.

**Objective:**

We sought to determine whether a dried tube specimen (DTS)-based PT programme would be a feasible option to support Xpert MTB/RIF EQA in RLS.

**Methods:**

Between 2013 and 2015, the United States Centers for Disease Control and Prevention developed and conducted a voluntary EQA programme using DTS-based PT material. Eight rounds of PT, each comprising five DTS samples, were provided to enrolled testing sites. After each round, participant results were compared to expected results, scored as satisfactory or unsatisfactory, and sites were provided with performance reports.

**Results:**

Programme enrolment increased from 102 testing sites in seven countries to 441 testing sites in 14 countries over the course of three years. In each PT round, approximately 90% of participating sites demonstrated satisfactory performance. In seven of the 14 enrolled countries, the proportion of sites with a satisfactory score increased between the first round of participation and the most recent round of participation.

**Conclusion:**

This programme demonstrated that it is possible to implement an Xpert MTB/RIF PT programme for RLS using DTS, that substantial demand for Xpert MTB/RIF PT material exists in RLS, and that country performance can improve in a DTS-based PT programme.

## Introduction

The World Health Organization recommends the Xpert^®^ MTB/RIF (*Mycobacterium tuberculosis*/rifampicin) assay (Cepheid, Sunnyvale, California, United States) as one of the initial diagnostic tests for all individuals evaluated for tuberculosis.^1,2^ The Xpert MTB/RIF assay is an integrated sputum processing and real-time polymerase chain reaction system that rapidly and simultaneously detects *M. tuberculosis* and the presence of mutations conferring resistance to rifampicin.^3^ The roll-out of the Xpert MTB/RIF assay to resource-limited settings (RLS) with a high tuberculosis burden has been remarkable. By the end of 2016, 6659 GeneXpert instruments had been purchased at concessional prices for use at multiple levels of the tiered laboratory network.^4^

External quality assessment is a critical component of a laboratory quality management system. Proficiency testing (PT) is one means of conducting external quality assessment for tests in clinical laboratories. Proficiency testing provides objective evidence of accurate testing and may identify areas in the diagnostic process, including pre-analytic, analytic, and post-analytic phases, where quality improvement is needed.^5,6^ The rapid scale-up of Xpert MTB/RIF testing in RLS has not coincided with the same investment in quality assurance activities for the assay. Few PT providers for the Xpert MTB/RIF assay offer materials to sites in RLS, leaving a substantial gap in external quality assessment implementation that could otherwise help to confirm site competency in assay administration and accuracy of test results for limited-resource countries.^7,8,9^

The United States Centers for Disease Control and Prevention (CDC) developed a PT panel for Xpert MTB/RIF, using a dried tube specimen (DTS)-based method.^10^ The DTS technique has successfully been used to develop PT panels for HIV, malaria and syphilis diagnostic assays in RLS.^11,12,13^ Our goal in this evaluation was to assess the performance and utility of CDC-prepared DTS panels in an Xpert MTB/RIF PT programme.

## Methods

### Ethical considerations

No patient specimen collection was required and no patient identifiers were recorded on quality assurance tools. Participation in the Xpert MTB/RIF quality assurance and PT programme was voluntary and free of charge. No incentives were provided. This study was approved by the CDC Center for Global Health Division of Human Research Protection (CGH HSR tracking number 2014-082).

### Dried tube specimen production

The method for producing DTS was described previously by DeGruy et al.^10^ Briefly, DTS were produced by chemically inactivating liquid cultures of well-characterised mycobacterial strains using equipment and supplies commonly found in laboratories conducting tuberculosis liquid culture.^14^ Mycobacteria were grown in BACTEC® MGIT 7 mL tubes (Becton, Dickinson and Company, Sparks, Maryland, United States) and inactivated using a 2:1 ratio of Xpert MTB/RIF sample reagent (Cepheid, Sunnyvale, California, United States) to MGIT culture incubated for 2 hours with intermittent vortexing. Inactivated cultures were washed, concentrated, resuspended, homogenised and diluted, before being dispensed into cryovials and air-dried in a Class II biological safety cabinet within a Biosafety Level III tuberculosis containment laboratory. Inactivation verification was performed by inoculating 0.5 mL of undiluted stock solution into MGIT tubes supplemented with polymyxin B, amphotericin B, nalidixic acid, trimethoprim, and azlocillin (PANTA) (Becton, Dickinson and Company, Sparks, Maryland, United States) and incubating for a total of 84 days over two incubation cycles in the BACTEC^®^ MGIT 960^®^ instrument (Becton, Dickinson and Company, Sparks, Maryland, United States) to confirm a lack of growth. Dried tube specimens were validated prior to distribution by testing 10% of DTS produced with the Xpert MTB/RIF assay (Cepheid, Sunnyvale, California, United States), as described previously.^10^ To test DTS, the DTS were rehydrated with 2.5 mL of sample reagent, shaken vigorously 20 times and incubated at room temperature for 15 minutes with additional shaking repeated after 10 min. The Xpert MTB/RIF cartridges were inoculated with approximately 2.0 mL of rehydrated samples using the transfer pipette provided with the kit, and tested immediately on a GeneXpert IV or GeneXpert VIII using GeneXpert DX software version 4.0 (Cepheid, Sunnyvale, California, United States) according to manufacturer instructions.^15^

### Proficiency testing programme

In 2013, select countries in sub-Saharan Africa, Southeast Asia, and the Caribbean, supported by the United States President’s Emergency Plan for AIDS Relief were invited to participate in CDC-Atlanta’s pilot DTS Xpert MTB/RIF PT programme at no cost. Seven countries elected to participate in the pilot, designated a PT country coordinator, and enrolled testing sites that routinely conducted Xpert MTB/RIF testing on patient specimens. These countries continued to enrol additional sites throughout 2013, and four additional countries enrolled in subsequent 2013 PT rounds. All countries remained enrolled throughout 2014. In 2015, enrolment was extended to additional sites in all participating countries plus three new countries in Africa.

The PT kits included a panel of five DTS samples, five transfer pipettes, testing instructions, and a paper form for recording results. Four PT rounds were distributed quarterly in 2013 (2013-A, 2013-B, 2013-C and 2013-D). Only one PT round was distributed at the end of 2014 (2014-A) to allow for evaluation of 2013 data. Three PT rounds were distributed in 2015 (2015-A, 2015-B and 2015-C). Country coordinators were responsible for distributing the panels to enrolled testing sites, collating results (including transferring data from paper forms to a template Microsoft Excel file (Microsoft Corp, Redmond, Washington, United States) and submitting data to CDC-Atlanta via email within nine weeks of panel receipt. A sufficient number of PT kits were sent to enrolled countries to allow for distribution of one PT kit to each enrolled site; enrolled sites that did not submit results were categorised as enrolled but not participating in a given PT round.

Participant data were analysed by CDC-Atlanta using Microsoft Excel. A report was generated for each participating site; this compared the site’s reported results to expected results (CDC-Atlanta validation results) and included the consensus results from all the participating testing sites in all the ountries. Consensus results detailed the number of testing sites detecting and not detecting *M. tuberculosis* complex, detecting and not detecting rifampicin resistance, reporting indeterminate rifampicin resistance, reporting uninterpretable tests (error, invalid, or no result), and not reporting *M. tuberculosis* complex and/or rifampicin results for each sample in the panel. To establish a consensus result for each PT sample in the round, we required at least 80% of participant results for that sample to match the expected result. In the 2013 pilot phase, participating sites were provided with qualitative reports indicating overall concordance of submitted results with the expected results. Beginning in 2014, participating sites were assigned a quantitative score for each PT round. Scores were calculated by assigning a value of 20 points to each of the five DTS, for a total of 100 possible points. If the site’s qualitative *M. tuberculosis* complex and rifampicin resistance detection results matched the expected results for a given sample, 20 points were awarded. If either the *M. tuberculosis* complex detection result or the rifampicin resistance detection result did not match the expected results, zero points were awarded. If the *M. tuberculosis* complex detection result matched the expected result but the site’s rifampicin resistance detection result was indeterminate, 10 points were awarded. If a site reported an uninterpretable test, five points were awarded. If a site did not report a result, zero points were awarded. A total score of greater than, or equal to, 80 points was considered satisfactory for the PT round. For this evaluation, 2013 results were retrospectively scored to compare performance across all years of the programme.

To further investigate concordance and discordance of site results with expected results, additional analyses were conducted in SAS version 9.3 (SAS Institute, Cary, North Carolina, United States). Results were categorised as concordant if both the qualitative *M. tuberculosis* complex and rifampicin results matched the expected results. If discordant, results were categorised according to the type of discordance (false-positive *M. tuberculosis* complex detection, unsuccessful run, etc.). Tests were considered unsuccessful when the test was started, but a definitive result was not obtained due to an error, invalid result, power failure during testing, or similar problem. Tests were also considered unsuccessful if the site chose not to test the sample, was unable to test the sample due to unavailability of Xpert MTB/RIF kits, or failed to report either the *M. tuberculosis* complex or rifampicin detection result. Frequencies for results in each category were calculated by PT sample and in aggregate for all samples. The mycobacterial strain used to produce the PT sample and the mean cycle threshold (Ct) of probe A during validation of the PT sample were included in the analysis to look for trends between these variables and the amount and type of discordance observed. In line with previous studies, probe A was selected for most analyses as it was the first probe to reach the detection threshold.^7^

We conducted additional statistical analyses to test for an association between the mean Ct of probe A during validation and several types of discordance including false-negative *M. tuberculosis* complex detection, false-positive rifampicin resistance, and indeterminate rifampicin resistance. Logistic regression of the type of discordance on mean Ct of probe A was conducted using R glm (R Foundation for Statistical Computing, Vienna, Austria).^16^

## Results

**FIGURE 1:**
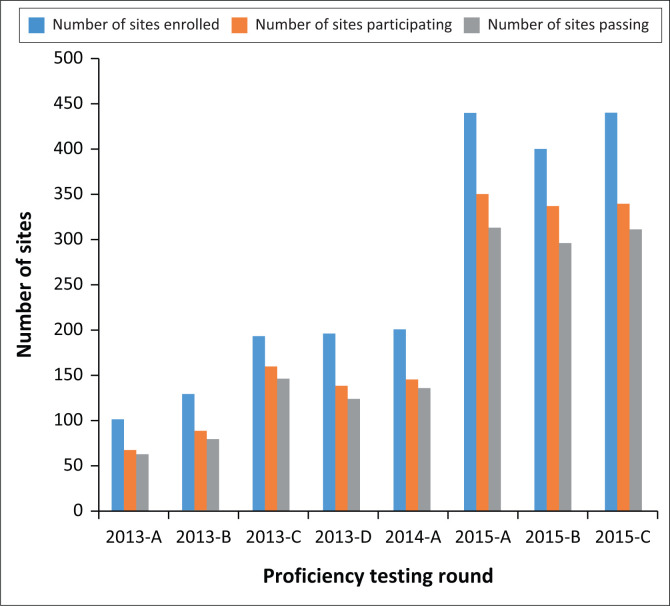
Number of sites enrolled, participating, and achieving a passing score in dried tube specimen-based Xpert^®^ MTB/RIF proficiency testing programme, 2013–2015.

Enrolment in the DTS-based Xpert MTB/RIF PT programme increased steadily from round 2013-A to round 2015-C, with 102 sites in seven countries enrolled in round 2013-A and 441 sites in 14 countries enrolled in round 2015-C (Figure 1). Participation by enrolled sites increased from 68 sites in six countries in round 2013-A to 342 sites in 13 countries in round 2015-C. The participation among sites ranged from a minimum of 66.7% in round 2013-A to a maximum of 83.8% in round 2015-B.

The proportion of participating sites with a satisfactory score ranged from a minimum of 88.1% in round 2015-B to a maximum of 93.1% in round 2014-A (Table 1). Individual countries had as few as 50% and as many as 100% of their participating sites achieve satisfactory scores. In seven of the 14 enrolled countries, the proportion of sites with a satisfactory score increased between the first round of participation and the most recent round of participation. Of the remaining seven countries, the proportion of sites with a satisfactory score remained constant in three countries but decreased in four countries (Table 1).

**TABLE 1a:** Number and percentage of sites participating and achieving passing scores in dried tube specimen-based Xpert® MTB/RIF proficiency testing programme for participating countries, 2013–2015.

Country	Proficiency testing round															
	2013-A				2013-B				2013-C				2013-D			
	No. participated/No. enrolled	%	No. passed/No. participated	%	No. participated/No. enrolled	%	No. passed/No. participated	%	No. participated/No. enrolled	%	No. passed/No. participated	%	No. participated/No. enrolled	%	No. passed/No. participated	%
1	0/12	0.0	NP	-	9/13	69.2	8/9	88.9	12/20	60.0	11/12	91.7	14/20	70.0	11/14	78.6
2	1/2	50.0	1/1	100.0	0/2	0.0	NP	-	2/2	100.0	2/2	100.0	4/4	100.0	4/4	100.0
3	21/26	80.8	20/21	95.2	16/26	61.5	14/16	87.5	21/26	80.8	20/21	95.2	12/26	46.2	11/12	91.7
4	8/12	66.7	7/8	87.5	8/12	66.7	4/8	50.0	10/12	83.3	9/10	90.0	0/12	0.0	NP	-
5	2/3	66.7	2/2	100.0	2/3	66.7	2/2	100.0	3/3	100.0	3/3	100.0	3/3	100.0	3/3	100.0
6	23/33	69.7	20/23	87.0	20/33	60.6	18/20	90.0	26/34	76.5	25/26	96.2	22/34	64.7	21/22	95.5
7	13/14	92.9	13/13	100.0	14/14	100.0	14/14	100.0	15/15	100.0	14/15	93.3	14/15	93.3	13/14	92.9
8	NE	-	-	-	NE	-	-	-	32/33	97.0	29/32	90.6	27/33	81.8	21/27	77.8
9	NE	-	-	-	12/16	75.0	11/12	91.7	8/16	50.0	8/8	100.0	14/16	87.5	14/14	100.0
10	NE	-	-	-	9/9	100.0	9/9	100.0	8/9	88.9	8/8	100.0	5/10	50.0	5/5	100.0
11	NE	-	-	-	NE	-	-	-	22/23	95.7	18/22	81.8	23/23	100.0	21/23	91.3
12	NE	-	-	-	NE	-	-	-	NE	-	-	-	NE	-	-	-
13	NE	-	-	-	NE	-	-	-	NE	-	-	-	NE	-	-	-
14	NE	-	-	-	NE	-	-	-	NE	-	-	-	NE	-	-	-

**Total for round**	**68/102**	**66.7**	**63/68**	**92.6**	**90/130**	**69.2**	**80/90**	**88.9**	**159/194**	**82.0**	**147/159**	**92.5**	**138/196**	**70.4**	**124/138**	**89.9**

NP, no participation; NE, not enrolled.

**TABLE 1b:** Number and percentage of sites participating and achieving passing scores in dried tube specimen-based Xpert® MTB/RIF proficiency testing programme for participating countries, 2013–2015.

Country	Proficiency testing round															
	2014-A				2015-A				2015-B				2015-C			
	No. participated/No. enrolled	%	No. passed/No. participated	%	No. participated/No. enrolled	%	No. passed/No. participated	%	No. participated/No. enrolled	%	No. passed/No. participated	%	No. participated/No. enrolled	%	No. passed/No. participated	%
1	13/20	65.0	12/13	92.3	33/34	97.1	29/33	87.9	34/34	100.0	29/34	85.3	34/34	100.0	32/34	94.1
2	6/6	100.0	6/6	100.0	11/11	100.0	10/11	90.9	11/11	100.0	11/11	100.0	11/11	100.0	11/11	100.0
3	7/26	26.9	7/7	100.0	51/57	89.5	46/51	90.2	55/57	96.5	52/55	94.5	47/57	82.5	45/47	95.7
4	12/12	100.0	12/12	100.0	32/36	88.9	26/32	81.3	33/36	91.7	26/33	78.8	33/36	91.7	24/33	72.7
5	0/3	0.0	NP	-	5/5	100.0	5/5	100.0	5/5	100.0	5/5	100.0	5/5	100.0	5/5	100.0
6	13/34	38.2	12/13	92.3	0/42	0.0	NP	-	25/42	59.5	18/25	72.0	26/42	61.9	25/26	96.2
7	18/18	100.0	18/18	100.0	25/26	96.2	24/25	96.0	24/26	92.3	22/24	91.7	24/26	92.3	24/24	100.0
8	33/33	100.0	29/33	87.9	41/42	97.6	41/41	100.0	41/42	97.6	36/41	87.8	33/42	78.6	30/33	90.9
9	13/16	81.3	13/13	100.0	47/52	90.4	42/47	89.4	36/52	69.2	32/36	88.9	33/52	63.5	27/33	81.8
10	8/10	80.0	6/8	75.0	13/18	72.2	10/13	76.9	11/18	61.1	10/11	90.9	15/18	83.3	12/15	80.0
11	22/23	95.7	20/22	90.9	34/39	87.2	32/34	94.1	NE	-	-	-	36/39	92.3	36/36	100.0
12	NE	-	-	-	11/12	91.7	8/11	72.7	11/12	91.7	9/11	81.8	12/12	100.0	12/12	100.0
13	NE	-	-	-	34/36	94.4	30/34	88.2	33/36	91.7	29/33	87.9	33/36	91.7	29/33	87.9
14	NE	-	-	-	14/31	45.2	11/14	78.6	18/31	58.1	18/18	100.0	0/31	0.0	NP	-

**Total for round**	**145/201**	**72.1**	**135/145**	**93.1**	**351/441**	**79.6**	**314/351**	**89.5**	**337/402**	**83.8**	**297/337**	**88.1**	**342/441**	**77.6**	**312/342**	**91.2**

NP, no participation; NE, not enrolled.

All 40 PT samples met the criteria for establishing a consensus result except 2015-B-1. 2015-B-1 was below 80% concordance with expected results, primarily due to a high rate of false-negative *M. tuberculosis* complex detection results (19.9%) (Table 2). This sample is further discussed in the Limitations section. For 29 of the 39 PT samples with a consensus result, over 90% of DTS results received were concordant with the expected result. Five of the 10 PT samples with < 90% concordance had false-negative *M. tuberculosis* complex detection as the most frequently observed cause for discordance, four of the 10 samples had unsuccessful tests (errors, invalids, and other causes) as the most frequently observed cause for discordance, while one sample had an equal number of unsuccessful tests and indeterminate rifampicin results. Error rates ranged from 0% to 5.9% across PT samples.

**TABLE 2:** Comparison of dried tube specimen results with expected results for Xpert® MTB/RIF proficiency testing programme, 2013–2015.

Proficiency testing round	Proficiency testing sample	Strain†	Expected result‡	Mean Ct of probe A during validation	Result category																		
					Concordant		False-positive MTB detection		False-negative MTB detection		False-positive RIF resistance		False-negative RIF resistance		Indeterminate RIF resistance		Invalid		Error		Unsuccessful test (other cause)		Total DTS results received
					*n*	%	*n*	%	*n*	%	*n*	%	*n*	%	*n*	%	*n*	%	*n*	%	*n*	%	
2013-A	1	KAN	*N*	N/A	63	92.7	1	1.5	0	-	0	-	0	-	0	-	0	-	4	5.9	0	-	68
	2	H526D	RR	22.5	58	85.3	0	-	3	4.4	0	-	1	1.5	1	1.5	0	-	4	5.9	1	1.5	68
	3	H37Rv	*T*	19.3	67	98.5	0	-	0	-	0	-	0	-	0	-	0	-	0	-	1	1.5	68
	4	S522L	RR	20.1	66	97.1	0	-	0	-	0	-	0	-	0	-	0	-	1	1.5	1	1.5	68
	5	S531L	RR	24.8	62	91.2	0	-	1	1.5	0	-	0	-	2	2.9	0	-	1	1.5	2	2.9	68
2013-B	1	3201	RR	22.4	86	95.6	0	-	0	-	0	-	2	2.2	0	-	1	1.1	1	1.1	0	-	90
	2	GOR	*N*	N/A	87	96.7	1	1.1	0	-	0	-	0	-	0	-	1	1.1	0	-	1	1.1	90
	3	BOV	*T*	24.7	80	88.9	0	-	2	2.2	2	2.2	0	-	3	3.3	0	-	1	1.1	2	2.2	90
	4	2116	RR	24.1	81	90.0	0	-	4	4.4	0	-	0	-	1	1.1	1	1.1	3	3.3	0	-	90
	5	H37Rv	*T*	22.9	80	88.9	0	-	4	4.4	2	2.2	0	-	1	1.1	0	-	3	3.3	0	-	90
2013-C	1	S522L	RR	22.3	135	84.9	0	-	10	6.3	0	-	3	1.9	5	3.1	1	0.6	5	3.1	0	-	159
	2	3223	RR	20.5	148	93.1	0	-	4	2.5	0	-	0	-	5	3.1	1	0.6	1	0.6	0	-	159
	3	H37Rv	*T*	24.4	147	92.5	0	-	1	0.6	5	3.1	0	-	3	1.9	1	0.6	2	1.3	0	-	159
	4	MAR	*N*	N/A	153	96.2	3	1.9	0	-	0	-	0	-	0	-	2	1.3	1	0.6	0	-	159
	5	H526Y	RR	24.7	146	91.8	0	-	1	0.6	0	-	1	0.6	3	1.9	0	-	6	3.8	2	1.3	159
2013-D	1	35805	*T*	20.9	120	87.0	0	-	2	1.5	1	0.7	0	-	6	4.4	0	-	8	5.8	1	0.7	138
	2	8777	RR	24.9§	127	92.0	0	-	2	1.5	0	-	1	0.7	2	1.5	1	0.7	5	3.6	0	-	138
	3	H526D	RR	23.2	125	90.6	0	-	1	0.7	0	-	3	2.2	4	2.9	0	-	5	3.6	0	-	138
	4	35813	*T*	21.4	127	92.0	0	-	4	2.9	1	0.7	0	-	1	0.7	0	-	4	2.9	1	0.7	138
	5	KAN	*N*	N/A	131	94.9	2	1.5	0	-	0	-	0	-	0	-	1	0.7	2	1.5	2	1.5	138
2014-A	1	H37Rv	*T*	21.1	135	93.1	0	-	2	1.4	1	0.7	0	-	1	0.7	0	-	5	3.4	1	0.7	145
	2	FOR	*N*	N/A	134	92.4	3	2.1	0	-	0	-	0	-	0	-	0	-	6	4.1	2	1.4	145
	3	H526D	RR	22.7	132	91.0	0	-	1	0.7	0	-	5	3.4	1	0.7	0	-	5	3.4	1	0.7	145
	4	BOV	*T*	23.8	138	95.2	0	-	0	-	2	1.4	0	-	0	-	0	-	3	2.1	2	1.4	145
	5	35811	*T*	25.9	125	86.2	0	-	7	4.8	0	-	0	-	3	2.1	0	-	6	4.1	4	2.8	145
2015-A	1	KAN	*N*	N/A	332	94.6	3	0.9	0	-	0	-	0	-	0	-	0	-	10	2.8	6	1.7	351
	2	BOV	*T*	21.7	314	89.7	0	-	17	4.9	5	1.4	0	-	4	1.1	0	-	8	2.3	2	0.6	351
	3	H37Rv	*T*	19.3	332	94.6	0	-	7	2.0	3	0.9	0	-	0		0	-	6	1.7	3	0.9	351
	4	INT	*N*	N/A	325	92.6	7	2.0	0	-	0	-	0	-	0		1	0.3	9	2.6	9	2.6	351
	5	H526Y	RR	22.7	295	84.1	0	-	34	9.7	0	-	6	1.7	3	0.9	0	-	4	1.1	9	2.6	351
2015-B	1	2116	RR	26.2	248	73.6	0	-	67	19.9	0	-	2	0.6	2	0.6	0	-	12	3.6	6	1.8	337
	2	H37Rv	*T*	21.5	313	92.9	0	-	4	1.2	6	1.8	0	-	0	-	0	-	7	2.1	7	2.1	337
	3	3201	RR	23.0	272	80.7	0	-	25	7.4	0	-	9	2.7	15	4.5	1	0.3	7	2.1	8	2.4	337
	4	GOR	*N*	N/A	307	91.1	8	2.4	0	-	0	-	0	-	0	-	1	0.3	10	3.0	11	3.3	337
	5	H526Y	RR	21.9	294	87.2	0	-	13	3.9	0	-	3	0.9	8	2.4	0	-	11	3.3	8	2.4	337
2015-C	1	INT	*N*	N/A	327	95.6	3	0.9	0	-	0	-	0	-	0	-	0	-	4	1.2	8	2.3	342
	2	H37Rv	*T*	21.6	322	94.2	0	-	2	0.6	5	1.5	0	-	1	0.3	0	-	5	1.5	7	2.0	342
	3	H526Y	RR	21.1	313	91.5	0	-	3	0.9	0	-	7	2.1	4	1.2	0	-	7	2.0	8	2.3	342
	4	S522L	RR	19.7	323	94.4	0	-	3	0.9	0	-	2	0.6	0	-	0	-	6	1.8	8	2.3	342
	5	KAN	*N*	N/A	315	92.1	3	0.9	0	-	0	-	0	-	0	-	0	-	9	2.6	15	4.4	342

**Total**	**7385**	**90.6**	**34**	**0.4**	**224**	**2.7**	**33**	**0.4**	**45**	**0.6**	**79**	**1.0**	**13**	**0.2**	**197**	**2.4**	**140**	**1.7**	**8150**

† , Strains used: *Mycobacterium bovis* ATCC 35722 (BOV), *M. fortuitum* ATCC 6841 (FOR), *M. gordonae* ATCC 14470 (GOR), *M. intracellulare* ATCC 13950 (INT), *M. kansasii* ATCC 12478 (KAN), *M. marinum* ATCC 927 (MAR), *M. tuberculosis* H37Rv ATCC 27294, *M. tuberculosis* ATCC 35805, *M. tuberculosis* ATCC 35811, *M. tuberculosis* ATCC 35813.

CDC laboratory-derived *rpoB* mutants Ser522Leu (S522L), His526Asp (H526D), His526Tyr (H526Y), Ser531Leu (S531L).

WHO 2011 PT strains: 2116, 3201, 3223, 8777.

‡ , *N*, MTB not detected; *T*, MTB detected, RIF resistance not detected; RR, MTB detected, RIF resistance detected.

§ , Probe C was used for sample 2013-D-2 because probe A did not detect amplification in this isolate.

N/A, not applicable; Ct, cycle threshold; DTS, dried tube specimen; MTB, *Mycobacterium tuberculosis*; RIF, rifampicin; CDC, United States Centers for Disease Control and Prevention; WHO, World Health Organization.

**FIGURE 2:**
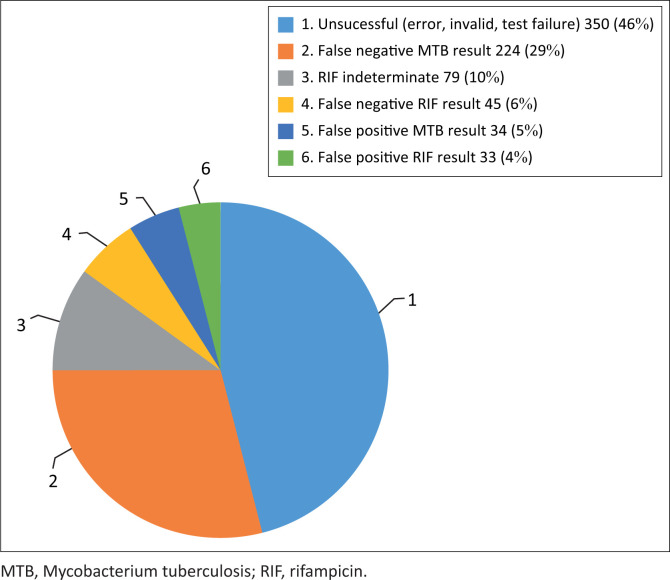
Distribution of 765 total discordant results in Xpert® MTB/RIF proficiency testing programme among participating countries, 2013–2015.

There were 765 (9.4%) discordant results out of 8150 DTS results received throughout the eight PT rounds. Of those, 350 (46%) discordant results were unsuccessful tests (Figure 2). False-negative *M. tuberculosis* complex results were the second-largest contributor to discordant results 224 (29%), followed by indeterminate rifampicin results 79 (10%). The remaining discordant results consisted of: false-negative rifampicin results 45 (6%), false-positive rifampicin results 34 (4%), and false-positive *M. tuberculosis* complex results 33 (5%).

Logistic regression of the proportion of false-negative *M. tuberculosis* complex detection results on the mean Ct of probe A during validation yielded an odds ratio of 1.42 (95% confidence interval [CI] 1.32–1.52). However, sample 2015-B-1’s unusually high mean probe A Ct, combined with its unusually high proportion of false-negative *M. tuberculosis* complex detection results, may have unduly influenced this estimate. When this sample was removed from the calculation, the odds ratio decreased to 1.14 (95% CI 1.04–1.26). Logistic regression of the proportion of indeterminate rifampicin results and the proportion of false-positive rifampicin resistance results on the mean Ct of probe A during validation yielded odds ratios of 1.14 (95% CI 1.02–1.25) for indeterminates and 0.96 (95% CI 0.79–1.16) for false positives.

## Discussion

We have shown that it is feasible to implement an Xpert MTB/RIF PT programme for RLS using DTS. Over the eight rounds of PT panels provided across a period of three years, the number of countries participating in the programme doubled and the number of participating sites increased five-fold. In each PT round, at least two-thirds of enrolled sites participated in PT panel testing and reported test results. Lower participation rates were seen in rounds during which certain countries or their enrolled sites were subsequently unable to participate due to logistical challenges, such as a lack of funding for in-country panel distribution, lack of staff, PT kits being lost in the mail between the country coordinator and the enrolled testing site, non-functioning Xpert instruments or computers, and stockouts (inability to procure test cartridges). Further work is needed to investigate and address the reasons for non-participation in proficiency testing as part of a comprehensive continuous quality improvement package.

Most participating sites performed well in the DTS-based Xpert MTB/RIF PT programme. Approximately 90% of participating sites in each PT round demonstrated satisfactory performance, and over 90% of returned results during the study period were concordant with expected results. The most common reason for a result that was not concordant with the expected result was an unsuccessful test. Some of the reported causes of unsuccessful tests, such as power failures and stockouts of testing kits, are recurring challenges in RLS.^17^

The use of a similar sample type, dried culture spots, for Xpert MTB/RIF external quality assessment in South Africa has been described.^7,8^ We observed similar error rates to those reported by Scott et al. and Gous et al.; however, we observed a greater proportion of discordant results.^7,8^ The average Ct of probe A obtained from dried culture spots was lower than that of many DTS PT samples. Lower Cts correlate with greater amounts of *M. tuberculosis* complex DNA present in the sample, indicating that a higher concentration of *M. tuberculosis* complex DNA was present in the dried culture spots. This may account for the smaller proportion of false-negative *M. tuberculosis* complex detection results from dried culture spots. We observed that false-negative *M. tuberculosis* complex detection results were often more common among DTS samples with higher probe A Cts during validation. Our analyses using logistic regression confirmed a statistically significant positive association between higher probe A Cts and false-negative *M. tuberculosis* complex detection. For DTS samples produced at CDC during the study period, an increase of 1 cycle in the mean probe A Ct resulted in an estimated 42% increase in the proportion of false-negative *M. tuberculosis* complex detection results (the estimate decreased to 14% when sample 2015-B-1 was excluded). Similarly, we found a statistically significant positive association between higher probe A Cts and indeterminate rifampicin resistance results, with an estimated 14% increase in the proportion of indeterminate rifampicin resistance results for every 1 cycle increase in a sample’s mean probe A Ct. No association was found between mean probe A Ct and false-positive rifampicin resistance, and we did not observe any trends between the strain used to produce DTS and the proportion and types of discordance during the study period. Based on these results, we are investigating modifications to the DTS preparation method that will increase the concentration of *M. tuberculosis* complex DNA present in DTS. However, it is not possible to rule out the presence of or determine the frequency of other site-specific factors, such as adherence to standard operating procedures, transcription errors, instrument maintenance and calibration, and DTS and test cartridge storage and shipping conditions that may influence the accuracy and reliability of Xpert MTB/RIF test results.

The large increase in enrolment we observed over the course of three years may have been influenced by several factors. As the World Health Organization endorsed Xpert MTB/RIF in 2010 and funds became available for the purchase of instruments, the number of laboratories in RLS utilising Xpert MTB/RIF increased dramatically.^4^ The World Health Organization’s expanded recommendations for use of Xpert MTB/RIF in 2013 also encouraged rapid adoption of the technology.^1^ Over the same time period, the number of laboratories in RLS electing to pursue external accreditation also increased, facilitated by such programmes as Strengthening Laboratory Management Toward Accreditation, and Stepwise Laboratory Improvement Program Towards Accreditation.^18,19^ Thus, an increasing number of laboratories in RLS are in need of Xpert MTB/RIF PT material to demonstrate proficiency in line with recommendations and requirements for continuous quality improvement and accreditation programmes.

### Limitations

Several challenges arose during operation of the DTS-based Xpert MTB/RIF PT programme. The participant consensus for sample 2015-B-1 was below 80% concordance with expected results, mainly due to false-negative *M. tuberculosis* complex results (failure to detect *M. tuberculosis* complex when the expected result was ‘MTB detected’). Although this sample was produced using the same method as other DTS samples, the probe A Cts during validation were on average higher than those of other *M. tuberculosis* complex samples produced. This indicates 2015-B-1 contained fewer inactivated organisms and thus less DNA than other samples, potentially contributing to the higher proportion of false-negative *M. tuberculosis* complex results received.^20^ To investigate the low concordance of 2015-B-1, three remaining aliquots were tested and confirmed the lower than average semi-quantitative result. Due to the low participant consensus, the decision was made to award all sites that submitted results for 2015-B-1 full credit (20 points). Based on this experience, we have implemented additional DTS validation criteria to ensure DTS contain enough inactivated organism to perform reliably. Beginning in 2016, only DTS samples with an average Ct value of less than 23 for the first probe detected are included in the distributed PT panels. The mean Ct value of 23 was selected based on the findings of previous investigators, who observed good concordance with mean Ct values up to 23.^7^ In addition, recording and reporting of results was also a recurring challenge. At times, no explanation was provided for missing or incomplete results, and it was not possible to determine whether a site attempted to test the sample or if the test was unsuccessful and the result not reported. More clearly defined reporting language and procedures were added to report forms in future PT rounds to reduce the reliance of free text data entry and improve our ability to provide assistance on determining the root causes of discordant results and unsuccessful tests.

### Recommendations and next steps

The steady increase in enrolment and number of sites participating in this DTS-based Xpert MTB/RIF PT programme demonstrates that there is a substantial demand for routine PT material for the Xpert MTB/RIF assay. The ease of large-scale batch preparation of DTS using readily available supplies and equipment, and the successful establishment of country coordinator roles for in-country management of PT programme operations, indicate that a DTS-based Xpert MTB/RIF PT programme could be a feasible option for countries wanting to manage their own Xpert MTB/RIF PT programme. Future work will focus on piloting the transfer of the DTS-based PT package to national and regional programmes as part of a comprehensive continuous quality improvement package, as well as developing a web-based PT data entry and reporting system.

### Conclusion

A continuous quality improvement package for Xpert MTB/RIF in RLS that includes routine PT material is an important contributor to ensuring provision of accurate results. A DTS-based PT programme for Xpert MTB/RIF is a useful tool for monitoring and improving Xpert MTB/RIF performance. The simplicity of producing DTS and use of common mycobacteriology laboratory supplies make DTS a feasible way for countries to provide Xpert MTB/RIF PT material to their testing sites.
